# Identification of Functional Domain(s) of Fibrillarin Interacted with p2 of *Rice stripe virus*

**DOI:** 10.1155/2018/8402839

**Published:** 2018-03-15

**Authors:** Luping Zheng, Jie He, Zuomei Ding, Chenlong Zhang, Ruoxue Meng

**Affiliations:** ^1^Key Laboratory of Biopesticide and Chemical Biology, Ministry of Education, Fujian Agriculture and Forestry University, Fuzhou 350002, China; ^2^College of Plant Protection, Fujian Agriculture and Forestry University, Fuzhou 350002, China

## Abstract

p2 of *Rice stripe virus* may promote virus systemic infection by interacting with the full length of fibrillarin from *Nicotiana benthamiana* (NbFib2) in the nucleolus and cajal body (CB). NbFib2 contains three functional domains. We used yeast two-hybrid, colocalization, and bimolecular fluorescence complementation (BiFC) assays to study the interactions between p2 and the three domains of NbFib2, namely, the N-terminal fragment containing a glycine and arginine-rich (GAR) domain, the central RNA-binding domain, and the C-terminal fragment containing an *α*-helical domain. The results show that the N-terminal domain is indispensable for NbFib2 to localize in the nucleolus and cajal body. p2 binds all three regions of NbFib2, and they target to the nucleus but fail to the nucleolus and cajal bodies (CBs).

## 1. Introduction

*Rice stripe virus* (RSV), an economically significant pathogen of rice, is the member of the genus *Tenuivirus*. It is transmitted by the small brown planthopper (*Laodelphax striatellus*) in a persistent, circulative-propagative manner, affected by global warming [1, 2]. *Nicotiana benthamiana* (*N. benthamiana*) can be infected by RSV through mechanical sap inoculation [3].

The genome of RSV comprises four single-stranded RNAs, denoted as RNA1, RNA2, RNA3, and RNA4 in the decreasing order of their molecular weights [4]. Exception is RNA1 that is negative sense and encodes only one protein responsible for viral replication; all the other three RNA segments employ an unusual ambisense coding strategy and encode two proteins: one in the viral-sense RNA (vRNA) and the other in the viral complementary-sense RNA (vcRNA) [5, 6]. RNA2 encodes two nonstructural proteins p2 and pc2; p2 is a viral RNA-silencing suppressor and is involved in systemic viral movement by interacting with fibrillarin [7, 8], and pc2 shares many characteristics common to the glycoproteins [9, 10]. RNA3 encodes a nonstructural protein p3, another suppressor of gene silencing [11], and a structural protein pc3, which is a nucleocapsid protein connected with resistance to RSV [12, 13]. The nonstructural disease-specific protein (SP) and the movement protein pc4 are encoded by RNA4 [14, 15].

Many different viruses bind to the nucleolus to manipulate host-cell functions and recruit nucleoprotein to aid in virus infection. Fibrillarin, an important nucleolus protein, was reported to interact with viral proteins and regulate virus replication, movement, and so on. Fibrillarin from *Nicotiana benthamiana* (NbFib2) mediates assembly of *Umbravirus* ribonucleoprotein particles (RNPs), which are capable of long-distance movement and causing systemic viral infection [16]. Protein 1 (NS1) in *Influenza A H3N2 subtype virus* binds to the fibrillarin via the C-terminal nuclear localization signal 2 (NLS2) [17]. There is also a close relationship between fibrillarin and suppressors of gene silencing. For example, fibrillarin interacted with viral genome-linked protein (VPg) in *Potato virus A* (PVA) and the 2b silencing suppressor protein in *Cucumber mosaic virus* (CMV), respectively [18, 19]. In our previous works, we found that p2 of RSV targeted to NbFib2 to promote virus systemic movement [8]. NbFib2 is an evolutionarily conserved protein, it is usually consisted of three domains, a glycine and arginine-rich domain (GAR), an RNA-binding domain, and an *α*-helical domain [20], but how p2 interacted with those motifs of NbFib2 is still unknown.

In this study, the interactions between p2 and the three domains of NbFib2 are identified using yeast two-hybrid, colocalization, and BiFC methods. The results reveal that p2 binds to the three domains of NbFib2 in the nucleus but fails to target to the nucleolus and cajal bodies (CBs), and the GAR domain is necessary for NbFib2 to localize in the nucleolus and CBs.

## 2. Results and Discussion

### 2.1. p2 Interacts with Three Domains of NbFib2 in Yeast Two-Hybrid Assay

**Figure 1:**
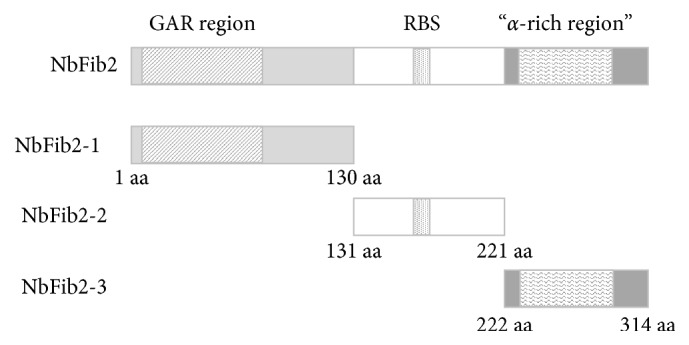
Three functional domains of NbFib2. NbFib2-1: N-terminal fragment from 1 aa to 130 aa, containing a GAR region and a glycine- and arginine-rich domain. RBS means RNA binding sites. NbFib2-2: the central RNA-binding domain from 131 aa to 221 aa. NbFib2-3: the C-terminal fragment from 222 aa to 314 aa, containing an *α*-helical domain.

**Figure 2:**
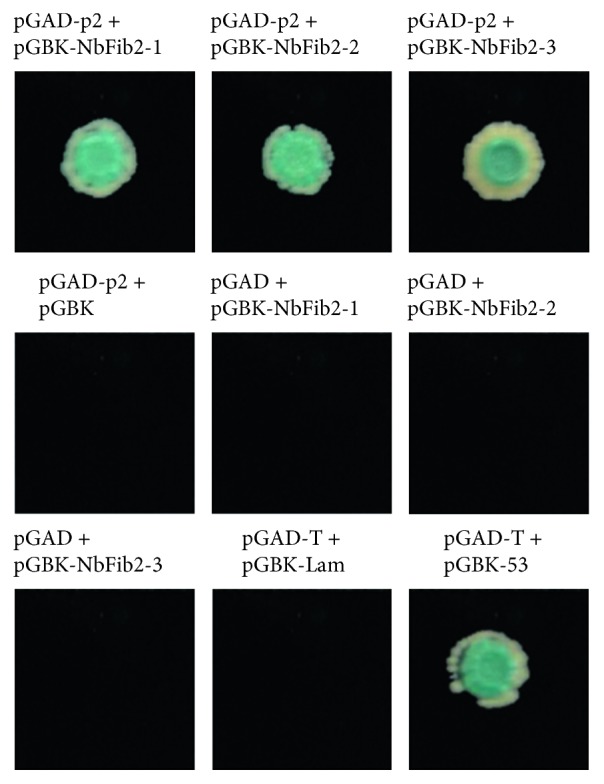
Interactions between p2 and the three functional domains of NbFib2 as examined by yeast two-hybrid assay. An X-*α*-gal assay shows that p2 interacts with the three domains of NbFib2, respectively. pGAD-T + pGB-Lam is a negative control, and pGAD-T + pGBK-53 is a positive control.

NbFib2 is composed of three functional domains: N-terminal fragment, containing a glycine and arginine-rich (GAR) domain (NbFib2-1), the central RNA-binding domain (NbFib2-2), and C-terminal fragment, containing an *α*-helical domain (NbFib2-3) (Figure 1). As shown in Figure 2, yeast cells cotransformed with pGADT7 (pGAD)-p2 and pGBKT7 (pGBK)-NbFib2 grew and turned blue on SD medium containing X-*α*-gal but lacking adenine (Ade), histidine (His), leucine (Leu), and tryptophan (Trp) (SD/Trp−Leu−His−Ade−/X-*α*-gal+), and the cotransformants of pGAD-T/pGBK-53 and pGAD-T/pGBK-Lam were individually used as positive control and negative control. However, the cotransformants of pGBK/pGAD, pGBK-NbFib2s/pGAD, pGBK/pGAD-NbFib2s, pGBK-p2/pGAD, or pGBK/pGAD-p2 failed to grow on SD/Leu−Trp−His−, although they grew well on SD/Trp−Leu− (Supplementary Figure S1). These results indicated that p2 of RSV interacts with the three domains of NbFib2 in yeast.

### 2.2. p2 Fails to Target to the Nucleus and Cajal Body in Colocation Assays

**Figure 3:**
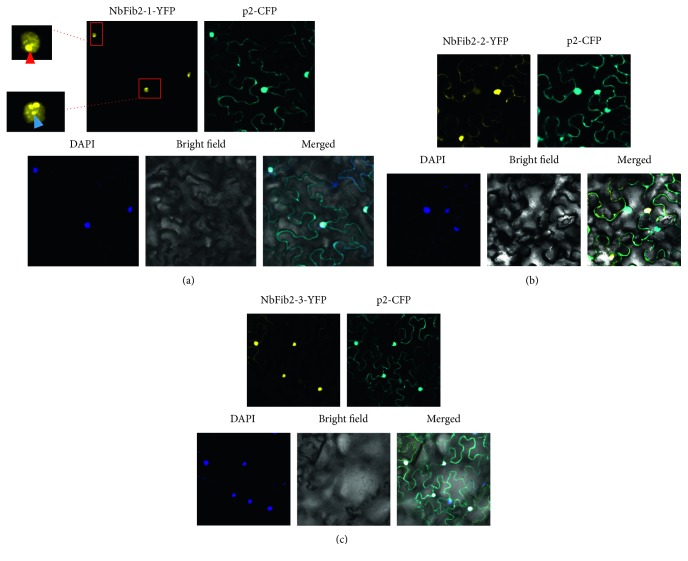
p2 colocalizes with the three functional domains of NbFib2 in the leaves of *N. benthamiana*. (a) p2-CFP was coexpressed with NbFib2-1-YFP. (b) p2-CFP was coexpressed with NbFib2-2-YFP. (c) p2-CFP was coexpressed with NbFib2-3-YFP. The nucleus was stained with 4′,6-diamidino-2-phenylindole (DAPI). Possible nucleolus and cajal body described in the text are designated with red and blue arrows, respectively. Fluorescence was observed at 48 h postinfiltration. Scale bars, 10 *μ*m.

Colocalization result shows that only NbFib2-1 (GAR domain) can form bright spots in the nucleolus and cajal body (CB) (Figure 3). The other two domains of NbFib2 (NbFib2-2 and NbFib2-3) also localize in nucleus, but they cannot agglomerate into small spots (Figures 3 and 3). p2 can colocalize with NbFib2-1, NbFib2-2, and NbFib2-3 in the nucleus but fail to form into granules in the nucleus and CB or in the cytoplasm (Figure 3).

### 2.3. p2 Binds Three Domains of NbFib2 in BiFC Assay

**Figure 4:**
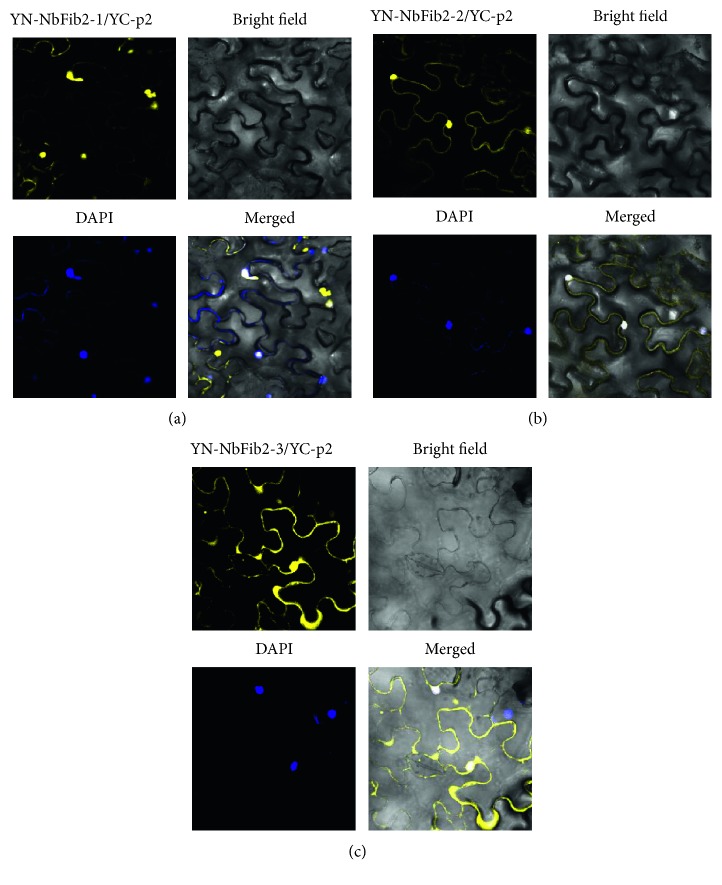
p2 interacts with the three functional domains of NbFib2 in BiFC assay. (a) YC-p2 was coexpressed with YN-NbFib2-1. (b) YC-p2 was coexpressed with YN-NbFib2-2. and (c) YC-p2 was coexpressed with YN-NbFib2-3. The nucleus was stained with 4,6-diaminophenylindole (DAPI). Fluorescence was observed at 48 h postinfiltration. Scale bars, 10 *μ*m.

In BiFC assay, p2 binds NbFib2-1, NbFib2-2, and NbFib2-3 individually, and they almost localize in the nucleus but not in the nucleus and CB in the shape of spots (Figure 4).

In summary, we found that (i) GAR domain was essential for NbFib2 to target to the nucleolus and CB, (ii) p2 interacted with the three functional regions of NbFib2, and (iii) these interactions occurred in the nucleus but failed to form bright spots targeting to the nucleolus and CBs.

NbFib2 is divided into three functional domains in our study; those domains localize in the nucleus, but only the N-terminal (GAR) domain targets to the nucleolus and CB as same as the full length of NbFib2. The GAR domain might be important for fibrillarin accumulation in the nucleolus [21]. It was reported that the GAR region was necessary and sufficient to target fibrillarin 1 from *Arabidopsis* and human cell to the nucleolus and CBs [22, 23]. Some findings demonstrate that the C-terminal region of fibrillarin targets it to CBs [23, 24]. However, our colocalization result shows that YFP of C-terminal region fails to accumulate and forms multiple spots in CBs. Thus, GAR domain is significant and indispensable for NbFib2 to target to the nucleolus or CB.

In this study, p2 binds to the three functional regions of NbFib2 in the nucleus but not in the nucleolus, CBs, or cytoplasm. These results are consistent with our previous studies, which show that p2 interacts with the full length of NbFib2, and NbFib2 plays a role in both the nucleolar localization and the appropriate cytoplasmic distribution of p2 [8]. The N-terminal fragment of fibrillarin, containing the glycine- and arginine-rich (GAR) domain, is supposed to be responsible for the interaction with various cellular and viral proteins, such as survival motor neuron (SMN), nucleocapsid protein of *porcine reproductive and respiratory syndrome virus* (PRRSV), and ORF3 of *groundnut rosette virus* (GRV) [25–27]. Our assays demonstrate that the GAR domain of NbFib2 is the region interactive with p2. p2 is an RNA-silencing suppressor (RRS); it may like other RRSs (p19 and HC-Pro) inhibit the intermediate step of RNA silencing via binding siRNA or the effector protein. Some indicate that fibrillarin is involved in the process of gene silencing induced by viruses, and fibrillarin interacts with long viral RNAs, rRNA, or siRNA [18, 28]. Fibrillarin 2 from *Arabidopsis* (AtFib2) has two RNA-binding regions, one located in the central region and the other located in the C-terminal region, while the GAR domain is incapable of RNA binding [28]. Fibrillarin is a highly conserved protein, and NbFib2 is highly homologous to AtFib2; thus, these two same domains of NbFib2 are capable of RNA binding. NbFib2-2 and NbFib2-3 may aid p2 to target to the siRNA or in other ways play a role in RNA silencing in plants.

In short, the results of this study are consistent with the previous study that the full length of NbFib2 is essential for p2 to target to the nucleolus and CBs. In addition, p2 interacts with the three functional regions of NbFib2, respectively; the mechanisms of these interactions will be studied in the future.

## 3. Materials and Methods

### 3.1. Plant Growth Conditions

The *N. benthamiana* plants were grown and maintained in a greenhouse at 25°C.

### 3.2. Plasmid Construction

cDNAs encoding the three domains of NbFib2 and RSV-p2 were amplified, respectively, by PCR using primers in Table 1, designed from *N. benthamiana* and RSV sequences (GenBank accession nos.: AM269909 and EF493228) downloaded from the GenBank. The three domains of NbFib2 were first inserted into the entry vector pDonr221 and then the destination vectors pEarleyGate101 (YFP), pEarleyGate201-YN (YN), and pEarleyGate201-YC (YC), using the Gateway recombination system [29]. pEarleyGate102-p2 (CFP-p2), YN-p2, and YC-p2 constructs were obtained by the same methods.

For yeast two-hybrid experiments, PCR products of RSV-p2 and the three domains of NbFib2 were digested with suitable restriction enzymes individually and then ligated to the vector pGADT7 or pGBKT7 digested with the same enzymes.

These constructs were confirmed by capillary sequencing conducted by Takara (Dalian, China).

### 3.3. Yeast Two-Hybrid Assay

pGBKT7-NbFib2s (three domains of NbFib2) were introduced together with pGADT7-p2 into the yeast strain AH109 by cotransformation. The cotransformants grew on different SD mediums: medium lacking tryptophan (Trp) and leucine (Leu) (SD/Trp−Leu−); medium lacking histidine (His), Trp, and Leu (SD/Trp−Leu−His−); and medium lacking adenine (Ade−), His, Trp, and Leu but containing X-*α*-gal (SD/Trp−Leu−His−Ade−/X-*α*-gal+). The cotransformation of pGADT7-NbFib2s and pGBKT7-p2 was also done the same way.

### 3.4. *Agrobacterium*-Mediated Transient Expression

*Agrobacterium tumefaciens* (*A. tumefaciens*) strain EHA105 were grown separately to OD_600_ = 0.8 at 28°C on the Luria–Bertani liquid medium supplemented with 50 *μ*g/*μ*L of rifampicin and 50 *μ*g/*μ*L of kanamycin. The resulting cultures were centrifuged at 12,000*g* for 1 min and then resuspended in induction media (10 mM MES, pH 5.6, 10 mM MgCl_2_, and 150 *μ*M acetosyringone). In colocalization and BiFC assays, *A. tumefaciens* containing NbFib2s were separately mixed with p2 in equal volumes. The mixtures of the bacterial cultures were incubated at room temperature for 3 h and then infiltrated onto fully-grown upper leaves. Six-week-old *N. benthamiana* was used for the experiment.

### 3.5. Confocal Imaging Analysis

Subcellular localizations of proteins were monitored at 48 h after infiltration under a confocal microscope (Leica TCS SP5, Leica Microsystems CMS GmbH). The fluorophores in CFP and YFP were excited at 458 and 514 nm, and images were taken using BA480–495 and BA535–565 nm emission filters, respectively.

## Figures and Tables

**Table 1:** The sequences, homologous recombination, and restriction sites of PCR primers.

Primer and purpose	Sequence (5′→3′)^a^	Modification
Construction for yeast two-hybrid assay		
*NS2-ADF*	CGggatccTGATGGCATTACTCCTTTTCA	*BamH1*
*NS2-ADR*	CCGctcgagTCACATTAGAATAGGACACTCAT	*Xho1*
*NbFib2-1-B DF*	GgaattcATGGTTGCACCAACTAGAGGTCG	*EcoR1*
*NbFib2-1-B DR*	CGggatccTCAGGGATTCCACACTCTGTATTCAACCT	*BamH1*
*NbFib2-2-B DF*	GgaattcATGCCGTTCTAAGTTAGCAGCTGCAGTT	*EcoR1*
*NbFib2-2-B DR*	CGggatccTCACACCATCCCGACAAGCATTCTG	*BamH1*
*NbFib2-3-B DF*	GgaattcATGGCCTGATCAGGCAAGAATTTTAGC	*EcoR1*
*NbFib2-3-B DR*	CGggatccTCAGGCAGCAGCCTTCTGCTTCTT	*BamH1*
Construction for entry vector pDONR221		
*NbFib2-GF*	ggggacaagtttgtacaaaaaagcaggcttcATGGTTGCACCAACTAGAGG	Homologous recombination
*NS2-GF*	ggggacaagtttgtacaaaaaagcaggcttcATGGCATTACTCCTTTTCA	Homologous recombination
*NS2-GR*	ggggaccactttgtacaagaaagctgggtcCATTAGAATAGGACACT	Homologous recombination
*NbFib2-1-G F*	ggggacaagtttgtacaaaaaagcaggcttcATGGTTGCACCAACTAGAGGTCG	Homologous recombination
*NbFib2-1-G R*	ggggaccactttgtacaagaaagctgggtcGGGATTCCACACTCTGTATTCAACCT	Homologous recombination
*NbFib2-2-G F*	ggggacaagtttgtacaaaaaagcaggcttcATGTTCCGTTCTAAGTTAGCAG	Homologous recombination
*NbFib2-2-G R*	ggggaccactttgtacaagaaagctgggtcCACCATCCCGACAAGCA	Homologous recombination
*NbFib2-3-G F*	ggggacaagtttgtacaaaaaagcaggcttcATGCCTGATCAGGCAAGAATT	Homologous recombination
*NbFib2-3-G R*	ggggaccactttgtacaagaaagctgggtcGGCAGCAGCCTTCTGCTTCTT	Homologous recombination

^a^The letters in lower case indicate homologous recombination sequence or a restriction enzyme site.

## Supplementary Materials

Supplementary Materials Figure S1: the cotransformants of yeast cells on SD/Trp−Leu−.
