# Supplementary figures and images for: Identification of Functional Domain(s) of Fibrillarin Interacted with p2 of Rice stripe virus
Source: Can J Infect Dis Med Microbiol. 2018 Mar 15;2018:8402839. doi: 10.1155/2018/8402839 (PMC5875058; doi:10.1155/2018/8402839)

## Supplements

Fig.S1 Co-transformants of yeast cells on SD/Trp-Leu-

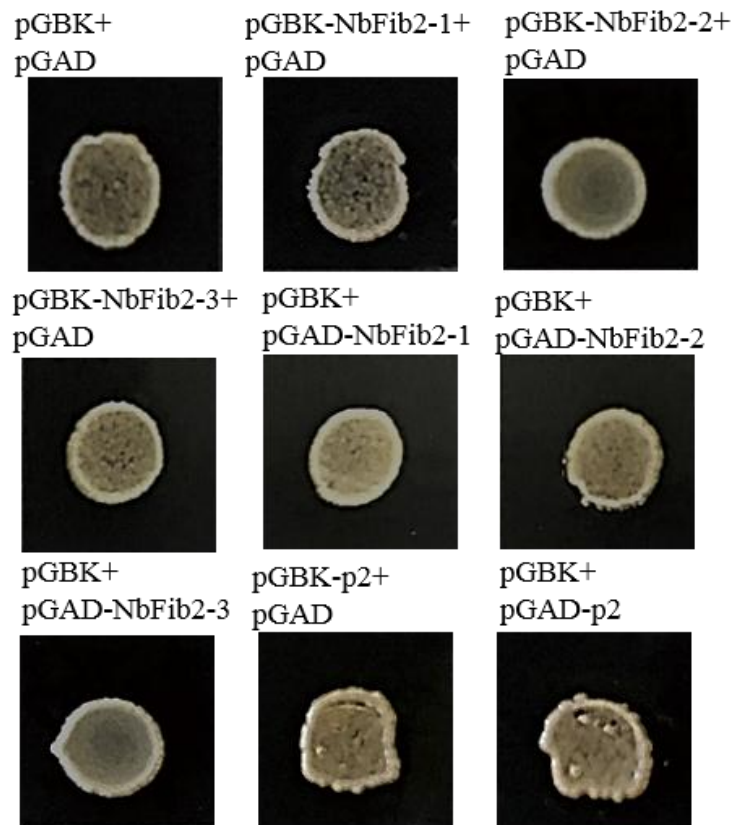

Supplement: Supplementary Materials — Figure S1: the cotransformants of yeast cells on SD/Trp−Leu−. [file 8402839.f1.pdf]
